# An imaging system for standardized quantitative analysis of *C. elegans *behavior

**DOI:** 10.1186/1471-2105-5-115

**Published:** 2004-08-26

**Authors:** Zhaoyang Feng, Christopher J Cronin, John H Wittig, Paul W Sternberg, William R Schafer

**Affiliations:** 1 Division of Biological Sciences, University of California, San Diego, CA USA; 2 Division of Biology, California Institute of Technology, Pasadena, CA 91125 USA; 3 Howard Hughes Medical Institute, California Institute of Technology, Pasadena, CA 91125 USA

## Abstract

**Background:**

The nematode *Caenorhabditis elegans *is widely used for the genetic analysis of neuronal cell biology, development, and behavior. Because traditional methods for evaluating behavioral phenotypes are qualitative and imprecise, there is a need for tools that allow quantitation and standardization of *C. elegans *behavioral assays.

**Results:**

Here we describe a tracking and imaging system for the automated analysis of *C. elegans *morphology and behavior. Using this system, it is possible to record the behavior of individual nematodes over long time periods and quantify 144 specific phenotypic parameters.

**Conclusions:**

These tools for phenotypic analysis will provide reliable, comprehensive scoring of a wide range of behavioral abnormalities, and will make it possible to standardize assays such that behavioral data from different labs can readily be compared. In addition, this system will facilitate high-throughput collection of phenotypic data that can ultimately be used to generate a comprehensive database of *C. elegans *phenotypic information.

**Availability:**

The hardware configuration and software for the system are available from wschafer@ucsd.edu.

## Background

The nematode *Caenorhabditis elegans *is among the most widely studied genetically-tractable experimental organisms. *C. elegans *is a soil-dwelling animal with a relatively simple and extremely well characterized anatomy; an adult hermaphrodite, for example, contains exactly 959 somatic cells, each with an identified position, morphology and cell lineage. Because of its short generation time, amenability to germline transformation, and completely sequence genome, it is ideally suited for classical and molecular genetic analysis. In particular, since the *C. elegans *nervous system is simple and well-characterized (the identity and connectivity of each neuron is known), it has become a facile model for studying the molecular basis for nervous system function. Robust behavioral phenotypes have been described for many *C. elegans *behaviors, including locomotion, egg-laying, mating and feeding, and these phenotypes have proven extremely useful for the genetic dissection of key aspects of neuronal function such as synaptic release, sensory transduction, and neuromuscular signalling [1].

Historically, a major limitation of such neurobiological studies in *C. elegans *has been the lack of quantitative methods for the evaluation of behavioral phenotypes. For example, the phenotypes of many behavioral mutants, even those defective in key aspects of neuronal signal transduction, appear subtle to a real-time human observer, and are difficult to assay without time and labor-intensive analysis of video recordings [2-4]. Even the phenotypes of mutants with grossly abnormal behavior are difficult to characterize precisely by manual observation. For example, mutants with striking defects in locomotion (uncoordinated, or Unc mutants) are typically classified using qualitative terms such as "coiler", "kinker", "sluggish", "slow" and "loopy" [5,6]. Since these descriptions are imprecise and subjective, it is extremely difficult if not impossible to assess the phenotypic similarity between two mutants based solely on such characterizations. Another challenge occurs in the analysis of behaviors such as locomotion and egg-laying, which can fluctuate over long time scales or involve infrequently-occurring events that are difficult to evaluate through real-time observation [7]. Furthermore, the quantitative assays that have been used in *C. elegans *behavioral studies (e.g. [8]) generally differ from lab to lab, and this lack of standardization has made effective comparison of data collected by different researchers difficult.

To address these problems, we have developed an automated tracking and image analysis system for the quantification of *C. elegans *behavioral patterns. Using this system, it is possible to record the behavior of individual animals at high magnification over long time periods and to simultaneously quantify a large number of behaviorally-relevant features for subsequent analysis. This system has wide applications for the dissection of complex *C. elegans *behaviors, and will also make it possible to comprehensively classify the behavioral patterns of *C. elegans *mutants on a genome-wide scale. By making this system widely available to *C. elegans *neuroscientists, we intend to define a software architecture that can be continually optimized and upgraded to incorporate new parameters that are useful to worm researchers, as well as a hardware platform that can be expanded to provide additional mechanical capabilities for the research community.

## Methods

To effectively capture the locomotion behavior of a freely-moving worm, it is necessary to acquire a sequence of images from which the animal's position, speed and body posture at any given point in time can be derived. *C. elegans *are small (1 mm) animals which, in the laboratory, are normally cultured on agar plates covered with a lawn of the common laboratory bacterium *Escherichia coli. *Nematodes move using an approximately sinusoidal wave motion that is propagated along the anterior/posterior axis in the dorsal/ventral plane. On an agar plate, the animal will normally lie on either its left or right lateral surface, making the waveform associated with movement visible from above. When crawling at maximum speed, an adult nematode travels at a rate of approximately 500 μm/s; thus, under the relatively high magnification (40–50 X) required to measure detailed features of body posture the worm can quickly crawl outside the field of view. It is therefore necessary to incorporate into the imaging system a motorized stage that can automatically follow the animal's movements and keep it in the microscope's visual field.

**Figure 1:**
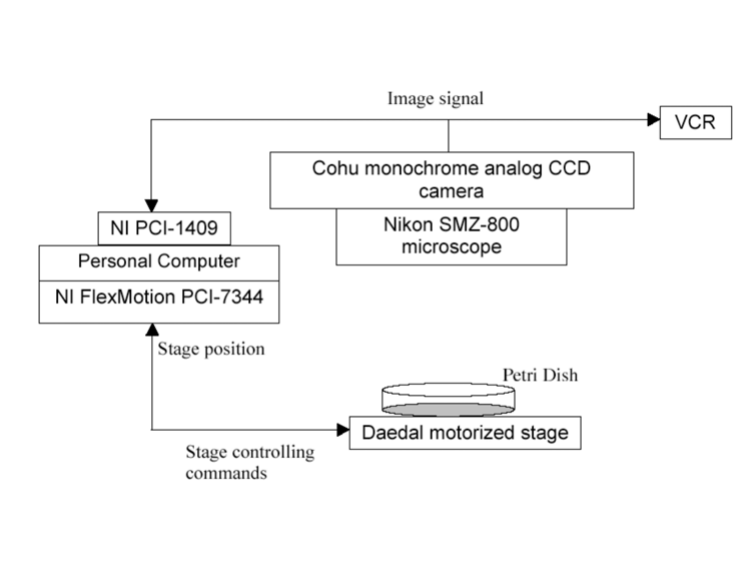
Illustration of the hardware of tracking system. A Nikon SMZ-800 microscope with a stereoscopic zoom is used to visualize the animals. Image signal can be simultaneously captured by a VCR and digitalized by a NI PCI-1409 image capture board through a Cohu monochrome analog CCD camera. NI FlexMotion PCI-1744 stage controller controls the Deadal motorized stage to follow the animal, and receives the stage position information.

The tracking system described here consists of (i) a Nikon SMZ-800 microscope with a stereoscopic zoom, for visualizing the animals; (ii) a Daedal motorized stage controlled by a National Instruments 4-axis controller, for maintaining the animal in the visual field; (iii) a Cohu monochrome analog CCD camera, for image acquisition; (iv) a Windows computer (PC) with a National Instruments video acquisition board, for tracking and image analysis (Figure 1). An optional VCR can also be included in the system for the purpose of cross verification of behavioral tracking. A complete parts list is in Additional file 1 ("hardware"). It should be noted that the software (see below) can, with very minor revisions, be adapted to other programmable motorized stages and frame grabbers that meet the industrial standards; questions about specific pieces of equipment can be addressed to the authors.

## Software

Briefly, the software for the system consists of four basic modules. The first module, called the tracker, allows the system to follow the worm as it crawls around the plate by directing the movements of a motorized stage to maintain the animal in the center of the field of view. As the video acquisition board acquires digital images from the microscope field, the tracker program identifies the animal from each acquired image based on 1. size of the objective isolated from background and 2. the direction of the animal crawling in previous frame if more than two objectives are found Based on the coordinates of the animal's centroid within the field of view, the tracker directs the movements of the stage to recenter the animal in the visual field when animals approach the edge of the image frame. The program then saves an image (460 × 380) of the worm containing visual frame (i.e, the pixels composing the worm body plus the minimum enclosing rectangle), the position of the animal within the field of view, the position of the stage, the time the image was captured, and other information crucial for behavioral analysis. These data are saved into the widely used .avi multimedia format, with a MPEG-4 filter to significantly compress the size of data. Null images are not saved into the .avi format, and the user is notified of the null frame. The highest frame rate with which the tracker can perform these operations with our current hardware setting is 30 frame/sec.

Next, the system contains a module (called the converter) that processes the raw images to simplify parameter estimation. First, the grayscale image is thresholded and converted to a binary image representing the worm outline. The image is further simplified by generating a morphological skeleton along the midline of each binary image and then distributing 30 skeleton points along this skeleton. A third module (called Lineup) then orders the backbone points from head to tail. To distinguish head and tail, minor user input is required to achieve 100% accuracy. In wild type, this user input (which involves identifying the head with a mouse click) is only required on 1% of the frames. Otherwise, all image processing is completely automated.

**Figure 2:**
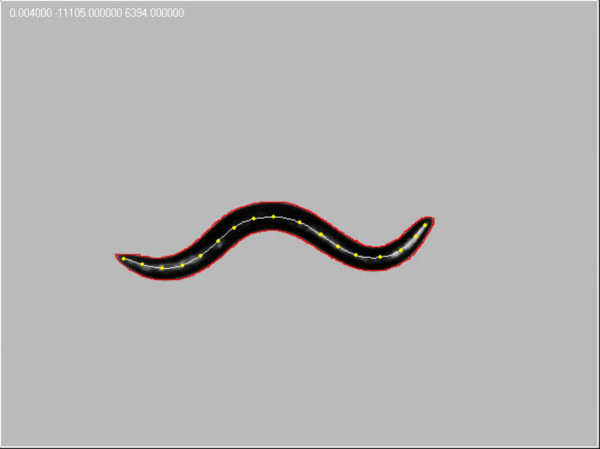
Representations of the *C. elegans *animal. The image is recovered from avi file. The Red line shows the boundary of the binary image. The skeleton is shown in white line and the yellow dots represent the skeleton points.

Thus, for each raw acquired image, the system generates 4 representations of the animal (Figure 2) of increasing complexity: the centroid representing the animal's position, the set of ordered skeleton points representing the animal's body posture, the binary image, which provides information about the size and shape of the animal, and the grayscale image, which retains information about the translucency of the animal. Together the outputs of the first three modules then are used to extract quantitative image features that define the characteristic behavioral pattern of a particular mutant type. During image processing stage, aberrant frames (e.g. containing a 'worm' with suddenly abnormal length) are marked and removed, and the user is notified of the defective image.

To obtain this information, the system has a fourth, parameter estimation module (called miner) that measures specific features based on grey/binary image, centroid, or skeleton point analysis that define important parameters related to locomotion or morphology. Broadly speaking, these include measurements of morphology, body posture, movement, and locomotion waveform. Morphological features include measurements of size, length, transparency, and elongation/eccentricity. Body posture features include measurements of body curvature as well as the occurrence of specific postures such as coils and omega turns. Movement features include centroid-based measurements of global speed and direction, skeleton point-based measures of local movement, and the occurrence of directional reversals and large turns. Waveform features include measurements of the frequency and amplitude of body bends, the flex of the animal's body during the locomotion wave, and the frequency and magnitude of foraging movements by the animal's nose. A total of 59 distinct features (Table 1) are measured by the system. For most of these features, three statistics (top 5% as maximum, mean and lower 5% as minimum) are calculated for each recording, giving a total of 144 measured parameters. A list of all the features and the algorithms used to generate them are found in supplemental data [see Additional file 2 "algorithms"].

## Implementation

The software is available in a PC version (compiled and benchmarked on a PC with 1 G Hz Pentium-III running Windows 2000 or XP). Software is written with C/C++, Labview 7.0 and Matlab (release 13), and complied with NI LabWindow 7.0. Installation disk and dataset samples are available upon request for non-profit academic usage with a license fee ($75, charged by National Instruments for the usage of their vision library; see Additional file 3 and 4, "codes" and "filelist" for details). Worm behavioral image data are in AVI format with a standard MPEG-4 filter (Microsoft MPEG-4 v2). Quantitative morphological and behavioral data are outputted into two widely distributed formats: Microsoft Excel and Microsoft Access. Using this hardware configuration, it is possible to process a 2 Hz 1 min real time data set in less than 5 minute (from image data to final data). Thus, it is feasible to envision using the system to screen for specific behavioral phenotypes among mutagenized *C. elegans.*

## Applications

We describe here a prototype for a standard, open-source system for automated phenotypic analysis of *C. elegans *behavior. We anticipate that such a system will be extremely useful to *C. elegans *neurobiologists, as machine vision offers a number of clear advantages over real-time observation for the characterization of behavioral phenotypes. First, it provides a precise definition of a particular mutant phenotype, facilitating quantitative comparisons between different mutant strains. For example, the waveform parameters have provided detailed information about the effects of neuronal G-protein signalling pathway genes on locomotion behavior. Even phenotypes that are extremely difficult to distinguish by eye (e.g. those of the calcium channel mutants *unc-2 *and *unc-36*) can be identified with relatively high reliability using the system [9]. In addition, it has been possible to use our system to reliably score behavioral events without labor-and time-intensive (and potentially biased) human scoring; for example, our system has been used to automatically detect directional reversals with high reliability in a touch avoidance assay [10]. Other specific postures such as coils can also be detected with high (>90%) reliability (Z. Feng, unpublished data).

With appropriate controls, a standardized phenotyping system also makes it possible to compare behavioral data collected by different researchers in different labs with greater precision than is possible using qualitative observer-driven approaches. In particular, a computerized system makes it possible to comprehensively assay multiple aspects of behavior simultaneously, yielding a complex phenotypic signature that can be used for bioinformatic studies [11]. In the future, we hope to use the tools described here to generate a comprehensive *C. elegans *phenotypic database that could be used to explore the clustering and relative similarities of mutant phenotypes.

## Supplementary Material

Additional File 1 Hardware list Click here for file

Additional File 2 Algorithms for feature measurements Click here for file

Additional File 3 Codes Click here for file

Additional File 4 File list Click here for file

## Figures and Tables

**Table 1:** Features measured by the automated system. List of the behavioral and morphological features. Detail algorithms to output these features are found in supplemental data. 144 statistical results (mean, maximum and minimum where applicable) of these features are output into Microsoft Access, while the values of these feature at each time when an image is grabbed are saved in Microsoft Excel.

Features	Comments	Catalog
Area	Total area of worm	Body morphology
BendingFrequency	Frequency of body bends	Wave form/speed
CmptFactor	Compactness factor	Body posture
ElgFactor	Elongation factor	Body posture
EllMAxis	Best-fit ellipse, major axis	Body posture
EllRatio	Best-fit ellipse, major axis/minor axis	Body posture
EqvlEllRatio	Equivalent ellipse ratio	Body posture
Fatness	Area/Length	Body morphology
Flex	Maximum skeleton point angle difference	Wave form
Foraging	Frequency of sideways (foraging) head movements	Specific behavior (feeding)
Foraging angle	Angle of foraging movements	Specific behavior (feeding)
Foraging distance	Distance moved by head during foraging	Specific behavior (feeding)
FRE	Frequency of angle change between skeleton points	Wave form/speed
GlbMvScope	Maximum distance moved from starting point	Global movement
GlbSpd	Speed of the animal's centroid movement	Global movement
HdTlRatio	Ratio of head to tail movement speed	Head movement
Heywood	Heywood circularity factor	Body morphology
Hydraulic	Hydraulic radius	Body morphology
IXX	Inertia XX	Body morphology
IXY	Inertia YY	Body morphology
IYY	Inertia XY	Body morphology
LclSpd	Local movement speed	Local speed
Length	Distance from head to tail	Body morphology/posture
LengthToPixel	Length/number of pixels in skeleton	Body posture
Loop	Percentage of time worms coils their body	Specific behavior (coil)
MaxIntercept	Max intercept	Body morphology
MeanIntcptPpdcl	Mean perpendicular intercept	Body morphology
MostPopularArea	Mode of area	Body morphology
MostPopularSpeed	Mode of speed	Global speed
PercentageMPArea	Frequency of occurrence of modal area	Body morphology
PercentageMPSpeed	Frequency of occurrence of modal speed	Global speed
Pushing	Local body movement speed/centroid speed	Locomotion wave efficiency
RectBigSide	Minimum enclosing rectangle (MER) length	Body posture
RectRatio	Minimum enclosing rectangle length/width ratio	Body posture
Reversal	Percentage of time that a worm performs reversals	Specific behavior (escape)
ReversalCount	Number of reversal sessions that a worm performs	Specific behavior (escape)
ReversalDisAve	Average distance traveled backward in reversals	Specific behavior (escape)
SktAglAve	Mean value of skeleton point vector angle	Body bending
SktCmptFactor	Length of worm / oriented MER area	Body posture
SktElgFactor	Skeleton enlongation factor	Body posture
SktHight	Oriented MER height	Body posture
SktIXX	Sum of x coordinate times x coordinates of skeleton points	Body posture
SktIXY	Sum of x coordinate times y coordinates of skeleton points	Body posture
SktIYY	Sum of y coordinate times y coordinates of skeleton points	Body posture
SktvAglAve	Average angle between skeleton points and centroid	Body bending
SktvAglMax	Max angle between skeleton points and centroid	Body bending
SktvDisAveToLength	Average distance between skeleton points and centroid/length	Body bending
SktvDisMaxToLength	Max distance between skeleton points and centroid/length	Body bending
SktvDisMinToLength	Min distance between skeleton points and centroid/length	Body bending
SktWidth	Oriented MER width	Body posture
Theta	Direction of centroid movement	Global movement
Thickness	Thickness of worm	Body morphology
TotalTravelDistance	Total distance traveled by a worm	Global movement
TrackAmplitude	Amplitude of waves in worm's track	Wave form
TrackWavlength	Wavelength of waves in worm's track	Wave form
Transparency	Transparency of worm body	Body morphology
Turn	Percentage of time that a worm performs a sharp turn	Specific behavior (search)
TypeFactor	Type Factor	Body morphology
Waddel	Waddel disk diameter	Body morphology
WaveLenth	Largest spatial span of a sine wave	Wave form
XSym	Sum of X coordinates of all sktps	Body bending
XYSym	Sum f Y coordinates of all sktps	Body bending
YSym	Sum of X coordinate * absolute value of Y coordinate	Body bending

## References

[B1] Rankin CH (2002). From gene to identified neuron to behaviour in *Caenorhabditis elegans.*. Nat Rev Genet.

[B2] Sawin ER, Ranganathan R, Horvitz HR (2000). *C. elegans *locomotory rate is modulated by the environment through a dopaminergic pathway and by experience through a serotonergic pathway. Neuron.

[B3] Brockie PJ, Mellem JE, Hills T, Madsen DM, Maricq AV (2001). The *C. elegans *glutamate receptor subunit NMR-1 Is required for slow NMDA-Activated currents that regulate reversal frequency during locomotion. Neuron.

[B4] Hardaker LA, Singer E, Kerr R, Zhou GT, Schafer WR (2001). Serotonin modulates locomotory behavior and coordinates egg-laying and movement in *Caenorhabditis elegans *. J Neurobiol.

[B5] Brenner S (1974). The genetics of *Caenorhabditis elegans *. Genetics.

[B6] Hodgkin J (1983). Male phenotypes and mating efficiency in *Caenorhabditis elegans *. Genetics.

[B7] Waggoner L, Zhou GT, Schafer RW, Schafer WR (1998). Control of behavioral states by serotonin in *Caenorhabditis elegans *. Neuron.

[B8] Pierce-Shimomura JT, Morse TM, Lockery SR (1999). The fundamental role of pirouettes in *Caenorhabditis elegans *chemotaxis. J Neurosci.

[B9] Geng W, Cosman P, Berry CC, Feng Z, Schafer WR (2004). Automatic tracking, feature extraction and classification of *C. elegans *phenotypes. IEEE Trans Biomed Eng.

[B10] Sanyal S, Wintle RF, Kindt KS, Nuttley WM, Arvan R, Fitzmaurice P, Bigras E, Merz D, Hebert TE, van der Kooy D, Schafer WR, Culotti JG, van Tol HHM (2004). Dopamine modulates the plasticity of mechanosensory responses in *C. elegans*. EMBO Journal.

[B11] Geng W, Cosman P, Baek JH, Berry CC, Schafer WR (2003). Quantitative classification and natural clustering of *C. elegans *behavioral phenotypes. Genetics.

