# Supplementary material for: An imaging system for standardized quantitative analysis of C. elegans behavior
Source: BMC Bioinformatics. 2004 Aug 26;5:115. doi: 10.1186/1471-2105-5-115 (PMC517925; doi:10.1186/1471-2105-5-115)
Supplement: Additional File 1 — Hardware list [file 1471-2105-5-115-S1.doc]

**Setting up an automatic *c. elegans* behavioral analysis system**

**Part I. Hardware needed**

**1. Motion system**

A) Stage

Component Manufacturer Model Number

4-axix controller [National Instruments](http://www.ni.com/) NI FlexMotion PCI-7344

Controller-Power National Instruments NI UMI-7764

Drive Interface

Interconnecting Cable (2 m) National Instruments NI SH68-C68-S

4” – Travel Precision Stage [Daedal](http://www.daedalpositioning.com/)  402LN Series Table

Stage (2)

Servo Motor with 10’ drive [Compumotor](http://www.compumotor.com/) ZETA Series 57-51

Interface cable (2) Servo Motor

Servo Drive Compumotor ZL4 Series Drive

Controller-to-Drive cable (2) Compumotor

Base Plate Daedal

XY Adapter Plate Daedal

B) Aluminum Petri dish holder

Component Mechanical design

Aluminum Petri Dish Holder [6cm plate](../6InchPlate.JPG)

(6cm plate)

Aluminum Petri Dish Holder [12cm plate](../12InchPlate.JPG)

(10cm plate)

**2. Vision System**

Component Manufacturer Model Number

Digital Camera Board National Instruments NI PCI-1428

Digital Camera [Cohu, co](http://www.cohu-cameras.com/). Sohu CMOS 7800

Camera link cable (3 m): Cohu, co.

Camera coupler [Diagnostic Instruments](../Diagnostic%20Instruments) HRP Video Couplers

2/3" Format (0.6x)

Micoroscope [Zeiss](http://www.zeiss.com/us/home.nsf) Stemi 2000-c

Light Source and Base [Mikron Instruments](http://www.mikcronet.com/)

**Part II. Hardware Assembly and Software Installation**

**1. Hardware assembly, vision/motion board driver installation**

The vendors provide detailed technical instructions for hardware installation and driver installation (if applicable), which should be sufficient for assembling the hardware of the tracking system. National Instruments, Inc. (<http://www.ni.com/support>) provides free technical support for motion/vision board installation.

Alternatively, [Viewpoint Systems, Inc.](http://www.viewpointusa.com/) provides assembling service at the cost of about $800.

**2. Software installation**

All software used in tracker system was developed by Tracker Group (Zhaoyang Feng, William Schafer, University of California San Diego, Christopher Cronin, Paul Sternberg, CalTech). National Instruments products (Programmer’s suite 7.0, NI Vision 6.0) and MathWork products (Matlab 6.3, Matlab compiler 6.3) are used in the programming. An installation disk is available upon request for non-profit academic usage with a license fee ($75, charged by National Instruments for the usage of their vision library).

A) Overview of the software packages

There are four software packages in the current tracker system: Tracker, Converter, Lineup and Miner.

Tracker is a computer program that controls the motion/vision hardware and tracks a single *c. elegans* animal movement. Program Tracker isolates animals from background and save image/stage data, time stamps, and other information crucial for behavioral analysis into a widely used multimedia format, avi, with a MPEG-4 filter to significantly compress the size of data. Depends on the computer and camera performance, the frame rate of the data collection varies. The highest frame rate with our current hardware setting is 30 frame/s. The resulted file is playable with a freely available multimedia player: Windows Media Player.

Converter is a computer program that performs the image processing to extrapolate the skeleton points for behavioral analysis from the avi files created by Tracker. The resulted data are automatically saved in files with extension name bkb. Converter is the most time-consuming step of the data processing for behavioral analysis. However, it takes less than one minute to process a five-minute video clip collected at 2 frame/s.

Lineup is a stands-along computer program. This program is used to identify the head and tail of animal automatically with minor human interfering. Lineup is the major computer program used to sort the head and tail of animals. After finishing the head/tail sorting, a file with extension name of bbs is created by Lineup.

Package miner contains two parts: Miner, a computer program extrapolating the features and a relational database that save the extrapolated data. Both data extrapolation and data saving are automatic. Miner can also be used to analyze behavior in detail.

B) Installation of software

To install the software packages, double click the setup.exe in the folder with the same name of the specific program and follow the prompts.

C) After program installation, created three folders under the root directory of disk c: ImageData, BackboneSet, wormExcelData, and wormDBase.

D) Copy file wormBehaviror.mdb (in installation disk) to folder, C:\wormDBase

E) Registration of database as following

- Click menu *Start::Settings::Control Panel*

- Click icon *Administrative Tools*

- Click icon *Data Sources (ODBC)*

- Click tab *User DSN*

- Click button *Add*

- Select item *Microsoft Access Driver (*.mdb)*

- Click button *Finish*

- Enter “wormBehaviror” under text box *Data Source Name*

- Click button *Select*

- Browse to c:\wormDBase

- Select file wormBehaviror.mdb

- Click button *OK*

- Click button *Advanced*

- Enter “beWorm” for *Login Name*, and “wurm” for *Password*

- Click button *OK*

- Click button *OK*

- Click button *OK*

G) Install the MPEG-4 filter by double click the file, MPEG4.exe in the install disk.

Protocols to use the software packages in tracker system

**Part I. Tracker**

1. Initiate and calibrate motion/vision systems.

A. If the system is assembled by [Viewpoint Systems, Inc.](http://www.viewpointusa.com/), the first step is to initiate the motion system using their software. To do so, start program , the initiation of motion system will start automatically. Sounds of motor should be heard. Click button *Exit* to terminate the program when calibration is done.

If the system is self assembled, contact Tracker Group to obtain initiate software and instruction.

B. After initiating the motion system, start the tracker program by double click Tracker.exe.

C. Click button *Initiate Tracker* to initiate the vision system.

D. Click button *Start Calibrate* and follow the prompts to calibrate the motion system.

E. Click button *Calibrate Optical System* and follow the prompts to calibrate the optical system. To gain best quality of data, the reading of text box *Background Value* should be within range of 175 ± 5. Otherwise, adjust the light source intensity or the reflection mirror of microscopy and repeat the optical calibration until the background reading is in the range.

Hardware initiation and calibration is only required once.

2. Setting parameter for tracking

There are six settable parameters are designed for specific experimental needs: frame rate (range 0 – 30; default value 2); auto timer (0 – 60 minutes; default value 30); maximum Area (range 3000 – 20000; default value 12000); minimum Area (range 500 – 4000; default 2500); maximum Length (range 50- 600; default value 450); minimum Length (range 0 – 100; default value 50).

The default values should work for most of the experiments. For animals with extreme size, try to play the last four changeable parameters to track the animals.

Theoretically, the frame rate can be set over 30 frame/s depends on the performance of the computer and camera. 30 frame/s is the upper limit of our current camera.

3. Tracking

Press cursors to find the animal and then click button *Save Binary* to start a tracking session. In the popup window, enter a file name and press button *OK*. To manually end a tracking session, click button *Write Binary Image File* again. To track animals without saving data, use button *Start Image & Tracker* instead of *Write Binary Image File*.

**Part II. Converter**

1. Start Converter by double clicking the executable.

2. Click button *Loading Image Files* to load the files that you want to convert.

3. In the popup window, select all the files the want to be converted.

4. Click button processing to batch process all the data automatically.

**Part III. Lineup**

1. Start Lineup and FrameByFrame.

2. Click button *Loading File* in Lineup to load a bkb file for lineup.

3. Click button *Loading Image Set* in FrameByFrame to load the corresponding AVI file.

4. Click button *Do All Lineup* in Lineup to start the automatic line up end point (head or tail).

5. If Lineup meets a difficulty, a window will pop up to ask user whether a switch is required.

6. If the original grey image is required to identify the head and tail, find the frame indexes in the text box *Which Node* and *Next Nod* in Lineup. Then choose the node index in FrameByFrame by setting the frame index, click button *Display Image Set Nodes*. The grey image with the interested index will display. Human eye is very sharp to identify the head and tail.

7. A message will popup when this step is finished. In this step, Click button *Sorting Head and Tail* to finalize the work.

8. Finally, click button *Save File* to save the data to a bbs file.

**Part IV. Miner**

1. Start Miner.

2. Click button *Load BBS Files* to load all the bbs files that you want to analyze.

3. Click button *Save Data* to batch process all the data.

Data will be automatically saved to excel files and database, which allows user to use commercial scientific software to explore behavioral features.

To use Miner to analyze the behavior, use following instructions.

3. Select the feature you want to explore, e.g. reversal validation by click the selection dropping lists under the corresponding catalog.

4. Activate cursor keys by clicking. Then use up and down cursor to explore data at an interesting time point. The values of all interested features are indicated by red cross hair at the corresponding figure fields. The centroid position of the animal at this given time is also indicted in figure *Tracker*.

5. To print and save an interesting figure, activate this figure by clicking it. Then press item *Print* in the menu.
