# Supplementary material for: An imaging system for standardized quantitative analysis of C. elegans behavior
Source: BMC Bioinformatics. 2004 Aug 26;5:115. doi: 10.1186/1471-2105-5-115 (PMC517925; doi:10.1186/1471-2105-5-115)
Supplement: Additional File 2 — Algorithms for feature measurements [file 1471-2105-5-115-S2.pdf]

Quantitative analysis of *C. elegans*: Algorithms to calculate behavioral and morphological features

Zhaoyang Feng[1], Christopher J. Cronin[2], Paul W. Sternberg[2,3], William R. Schafer[1]

1 Division of Biological Sciences, University of San Diego, California, CA 92122
2 Division of Biology, California Institute of Technology, Pasadena, CA 91125
3 Howard Hughes Medical Institute, California Institute of Technology, Pasadena, CA 91125

Basic concept of *C. elegans* abstractions used for quantitative analysis

Quantitative analysis using our system begins with video of subject worms. For our analyses video is treated as a sequence of grey-scale worm images which are used both as-captured and abstracted (using widely accepted image analysis theories and self-defined algorithms) to facilitate quantification of animal morphology and behavior. For the many attributes of interest we reduce each grey-scale worm image to a:

- a binary image representing 'worm' vs. 'background'
- a curve representing the worm's 'skeleton'
- a set of points ('skeleton points' or 'sktps') segmenting the worm's skeleton curve
- a single point representing the worm's centroid
- a line ('skewer fitting')

From these representations, as well as the original grey-scale images, our automated system quantitates features describing:

- morphology
- movement
- body bending (posture of one part of body to the rest)
- body waving (flexation of one part of body to the rest)

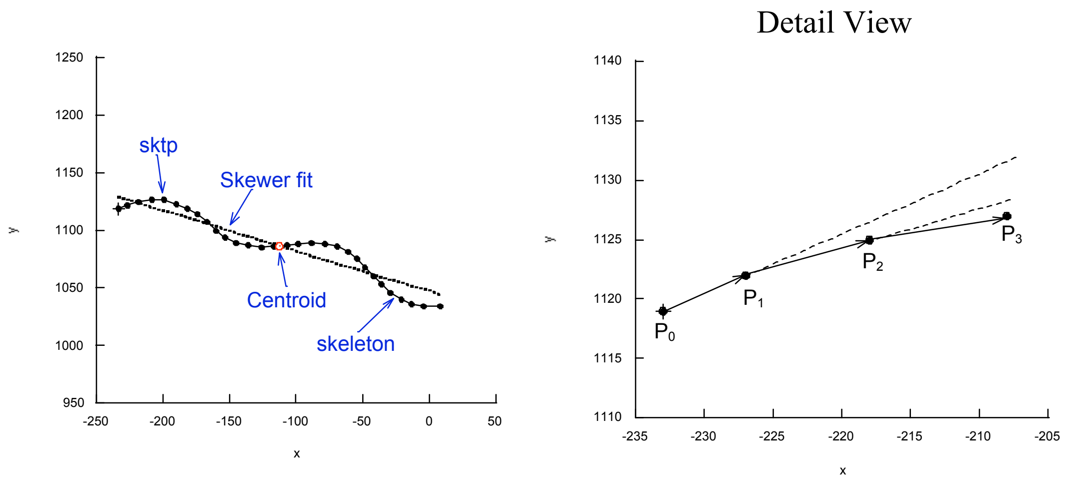

Figure 1. Left panel shows a wild type (N2) animal represented by its skeleton, a skeleton point set of 30 skeleton points, its skewer fitting, and its centroid in the Tracker field. Right panel is the detailed view of first four skeleton points ($P_0 - P_3$) in this skeleton point set.

Abstract representations:

Centroid: The centroid (shown as the red circle in the left panel of Figure 1) is the point whose coordinates represent the mean value of the coordinates of all points (pixels) representing 'worm' (as opposed to 'background') in a binary worm image.

Skeleton: A skeleton or 'skt' (shown as the solid curve in the left panel of Figure 1) is the curve resulting from mathematically thinning the binary image of the worm.

Sktp: The skeleton is segmented with a user-defined number of equally spaced dots with each dot defined as a skeleton point or 'sktp'. By our notation, the group of dots for a worm is a skeleton point set or 'sktp set'. The sktps in a sktp set are ordered with the sktp representing the head of the animal designated as P_0 and is marked by a cross (Figure 1). Sktp P_i is the ith sktp after P_0 along the animal's skeleton. We designate the vector from P_i to P_{i+1} as skeleton point vector[i] (sktp vector). Further, the angle from sktp vector[i] to sktp vector [i+1] is designated as sktp vector angle[i] (Right panel Figure 1).

Skewer Fit: A 'skewer fit' (the dashed line in the left panel of Figure 1) is the line that passes through the centroid and is parallel to a line linking the head and tail sktps.

Feature Quantification:

> Note: For length and distance measurements we use 'pixels' as our basic measurement unit where we consider a pixel to be the width or height of an individual square pixel in a worm image. The user must provide the appropriate scaling factors (based on their system's optics and image handling hardware/software) to convert pixels to an appropriate physical measurement like millimeters.

Morphology

1. Basic morphological features

Area	=	Number of pixels representing the animal in a binary image
Length	=	Length (in pixels) of the skeleton curve. (Note: 'Length' must not be confused with the number of square pixels needed to represent a contiguous curve in a real image, a number which will always be greater than or equal to the curve 'length.')
Thickness	=	Width (in pixels) of the worm at its midpoint. Measured as the length of the shortest line across a worm passing through the midpoint of the worm skeleton. (This line will typically be perpendicular to the worm skeleton at the skeleton midpoint.)
Transparency	=	Mean gray value of the animal in a grey-scale image after intensity calibration
Fatness	=	Area / Length
LengthToPixel	=	Length / number of pixels in a skeleton (see note above regarding pixels)
Loop	=	Percentage of time worms curls or 'loops.' Our automatic system distinguishes three types of loops:<br>Type 1: Worm head or tail sktp contacts another part of its body. (Consider the shape of a lowercase Greek letter 'delta.')<br>Type 2: Worm skeleton curve crosses itself. (Consider the shape of a lowercase Greek letter 'gamma.')<br>Type 3: Worm skelton forms a closed curve or spiral without crossing itself

2. Morphology based on binary image analysis
The following features are quantified using widely accepted image quantitative analysis algorithms provided in National Instruments' IMAQ Vision software (1999 edition). The titles of the features as referenced in our system and the corresponding National Instruments IMAQ features are listed below. The details of these morphological features are fully described in National Instruments' IMAQ Vision documentation.

**Our Feature**	**Feature in National Instruments' IMAQ Vision Quantitative Analysis documentation**
CmptFactor	Compact factor
ElgFactor	Elongation factor
EllMAxis	Ellipse major axis
EllRatio	Ellipse ratio
EqvlEllRatio	Equivalent ellipse ratio
Heywood	Heywood circularity factor
Hydraulic	Hydraulic radius
TypeFactor	Type Factor
IXX	Inertia XX
IYY	Inertia YY
IXY	Inertia XY
MaxIntercept	Max intercept
MeanIntcptPpdcl	Mean perpendicular intercept
RectBigSide	Rectangle big side
RectRaio	Rectangle ratio
Waddle	Waddle disk diameter

3. Morphological features based on sktp vectors
These features describe aspects of skeleton bending.

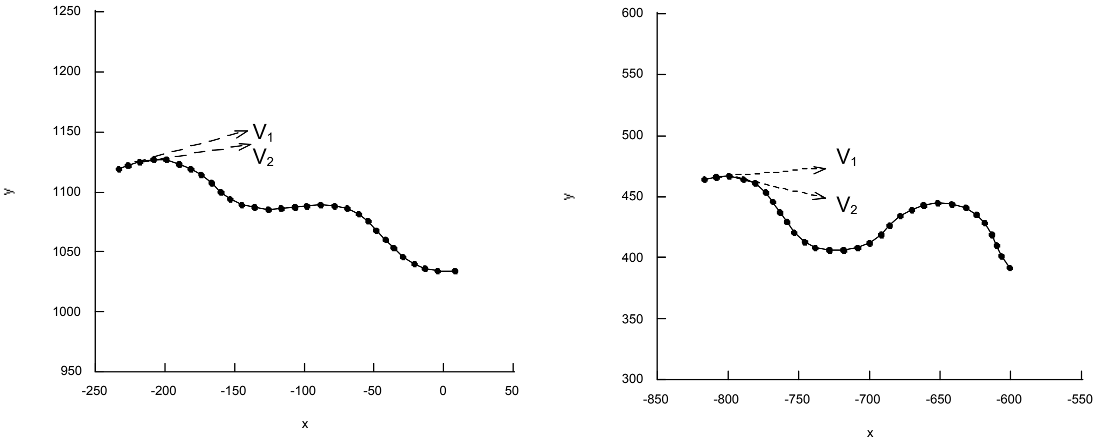

Figure 2. Two sktp sets from the same animal with consecutive sktp vectors V_1 and V_2 shown. Increased skeleton curvature in the right panel yields increased skeleton point vector angles compared to that those in the left panel. (Scales of axes in both panels are the same.)

SktvAlgAve	=	Mean value of sktpVector angle in a sktp set.
SktvDisAveToLength	=	Mean distance between adjacent skeleton points / skeleton length

SktvDisMaxToLength = Maximum distance between adjacent skeleton points / skeleton length
SktvDisMinToLength = Minimum distance between adjacent skeleton points / skeleton length
SktvAglMax = Maximum sktVector Angle in a sktp set.

Body posture

Our automatic system mathematically rotates and offsets the matrices of skeleton point set coordinates to measure aspects of body posture.

1. Body posture (Part I)

We rotate and offset the worm's skeleton point set to align the skewer fit to the x-axis with the centroid at the origin (X = 0, Y = 0) and with P_0 (the sktp representing the worm's head) as the smaller x-value. In this orientation we determine the size of the best-fit rectangle for the skeleton point set with sides parallel to the X and Y axes.

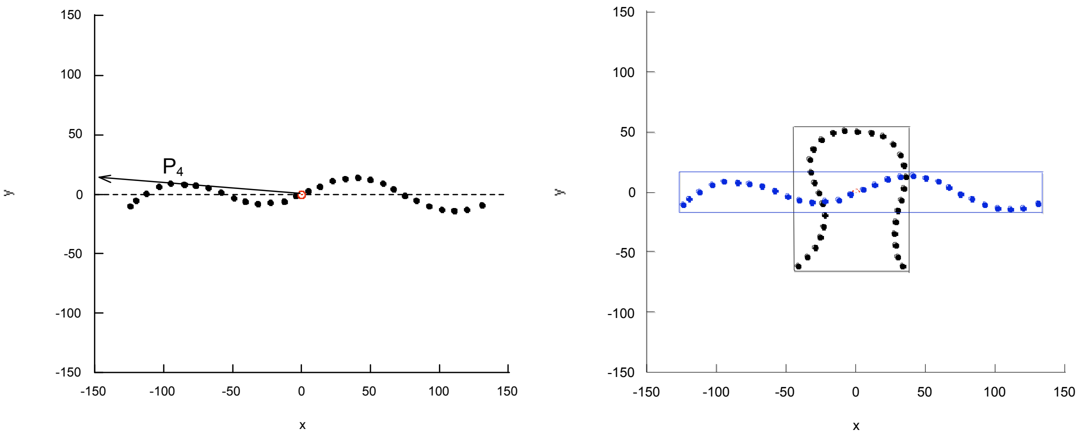

Figure 3. Left panel shows the same sktp set as in Figure 1 after the rotation. In right panel, this rotated sktp set (blue) is shown together with the rotated sktp set from the same animal performing an omega turn.

SktHight = Height of the bounding rectangle along the y axis
SktWidth = Width of the bounding rectangle along the x axis
SktCmptFactor = Skeleton 'compactness' factor, calculated as Skeleton length / bounding rectangle area
SktElgFactor = SktWidth / SktHight (the aspect ratio of the rotated worm)
SktIXX = Moment of inertia about the y-axis: [illegible]
SktIYY = Moment of inertia about the x-axis: [illegible]
SktIXY = Product of inertia: [illegible]

The rotated sktp set helps in automatically identifying omega turns, since the sktp set of an animal in an omega turn has a smaller SktElgFactor value and larger SktHight and SktAglAve values. (See below for detail algorithms of these features.) The features obtained from rotated sktp analysis may also be useful to quantify other morphological identifications.

Here we also introduce an additional measure, sktAgl, the angle between the skewer fit line and a line between a skeleton point and centroid (Figure 4) which we see as a means of quantifying an aspect of a worm's range of motion. With a worm's skeleton point set rotated and offset, sktAgl is the angular position of a skeleton point in polar coordinates. SktAglAve is the mean value of sktAgls in a sktp set.

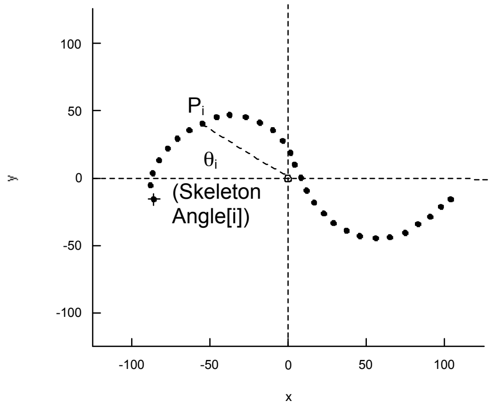

Figure 4. After the rotating a sktp set, the ith sktAgl [i] or θi is defined as the angle between the x-axis and the line linking the i'th sktp, P_i, and the centroid (origin).

sktAgl	=	Angle between the skewer fit line and the line linking the i'th sktp, P_i, and the centroid
SktAglAve	=	Mean value of sktAgl in a sktp set

2. Body posture (Part II)

A sktp set can also be rotated and offset so that the skewer fit is parallel to x-axis, the average of x, y maximum and minimum is the origin, and the sktp representing head of the worm on the left of y axis.

We also rotate and offset the worm's skeleton point set to align the skewer fit to the x-axis, but with the mean of the worm's rotated x- and y- maximum and minimum as the origin, again with P_0 (the sktp representing the worm's head) as the smaller x-value.

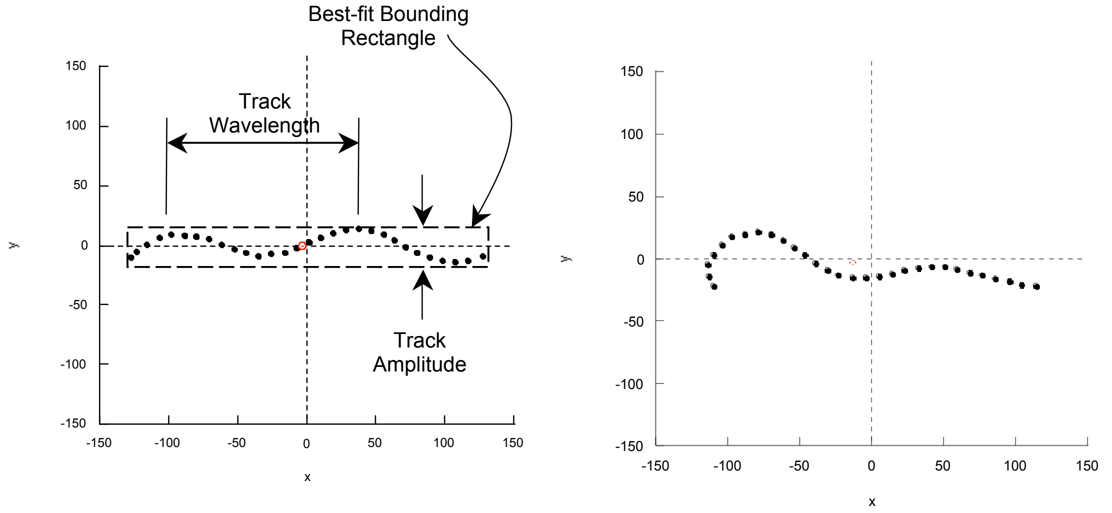

Figure 5. Left panel, the sktp set from Figure 1 rotated, depicting a worm in sinusoidal movement. Right panel shows a sktp set from an *unc-29* animal as an example of non-sinusoidal movement generating a larger wave at its head and with reduced wave amplitude along the rest of the body (possibly the marker of a kink). Left panel also shows track amplitude ('TrackAmplitude') and track wavelength ('TrackWavelength').

For an animal moving with a sinusoidal waveform, the sktp set representing this waveform should be symmetric in three ways on the rotated tracker field. First, the sum of x coordinates is zero, i.e. x-axis symmetric. Second, the sum of y coordinates is zero, i.e. y-axis symmetric. Finally, XYSYM (see below) is zero, i.e. it is symmetric against origin (left panel).

To calculate track wavelength, our system treats the coordinates of a rotated skeleton point set as a sinusoidal "signal." We perform a standard Fast Fourier Transform (using Matlab-provided algorithms) on the sinusoidal "signal," calculating in the spatial domain (as opposed to the time domain) which yields component frequencies in "cycles [or waves] per pixel" (that is, working in the spatial domain). Selecting the lowest non-zero frequency as the 'characteristic' frequency, we find the inverse to be the worm's wavelength in "pixels per wave" which is converted into convenient physical units (for example millimeters/wave) with the appropriate user-provided scaling factor.

XSYM	=	Sum of X coordinates of all sktps:
YSYM:	=	Sum of Y coordinates of all sktps.
XYSYM	=	Sum of (X coordinate * absolute value of Y coordinate).
TrackWavelenth	=	Wavelength of the sinusoidal shape of a rotated skeleton point set.
TrackAmplitude	=	Width of best-fit bounding rectangle for rotated skeleton point set.

Movement

1. Features based on centroid analysis

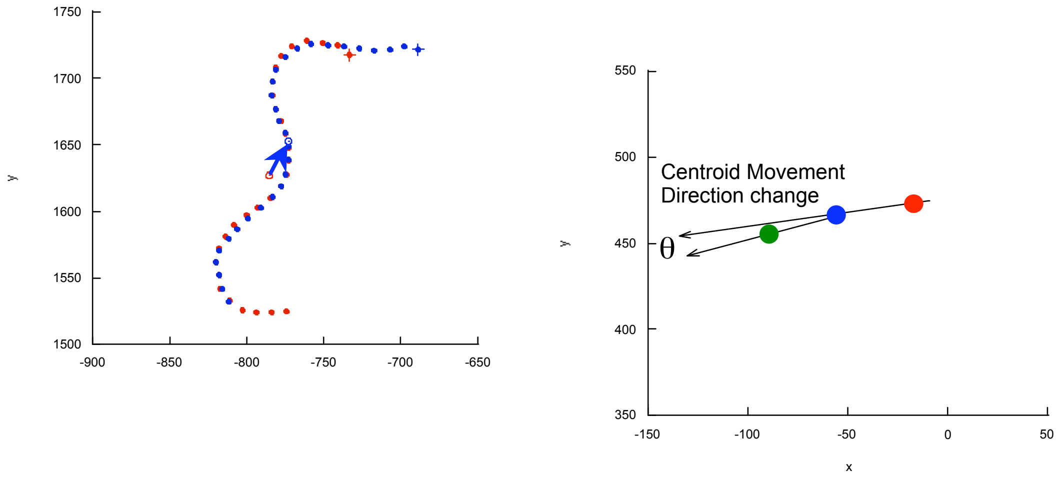

Figure 6. Left panel shows the sktp sets for an N2 animal at two times: time τ (depicted in red) and τ + 1 (depicted in blue), with the centroid for each sktp set depicted as a circle of matching color. The features analyzed by centroid movement are calculated from centroid coordinate changes. Right panel shows the centroid positions of an N2 animal at three consecutive times τ – 1 (red), τ (blue) and τ + 1 (green), depicting a change in worm centroid movement direction, Theta.

A centroid moving vector is defined as the vector from the centroid at time τ to the centroid at τ + 1.

TotalTravelDistance = Total distance traveled by a worm centroid during a tracking session

GlbSpeed = Centroid speed, calculated as distance centroid moves during a known time interval (the time between successive worm images).

GlbMvScope = Maximum of the straight-line distances between centroid position at any time during tracking session and centroid's initial position at time = 0.

Theta = Direction of each centroid movement vector during a tracking session

2. Features based on sktp analysis (Part I)

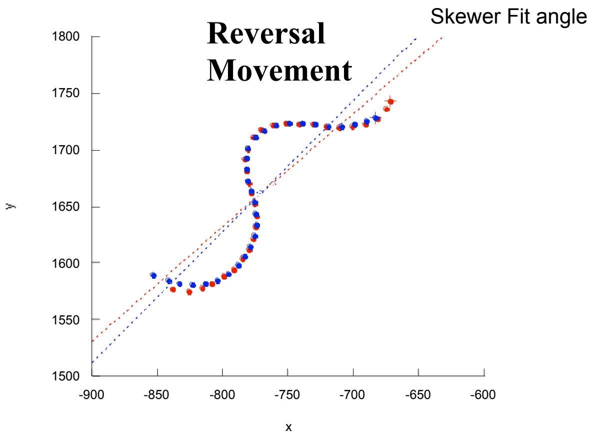

Figure 7 shows the sktp sets, centroids and skewer fits for an N2 animal at two times spaced 0.5 second apart while performing a reversal. (Time τ is in red, time τ + 0.5 second is in blue.)

When the angle between an animal's skewer fits for consecutive times is smaller than 10º, our system identifies a movement reversal provided the following conditions are satisfied after superimposing two sktp sets:

1) between time _ and _ + 1, the head skeleton point moves *toward* where the tail skeleton point was at time _
2) between time _ and _ + 1, the tail skeleton point moves *away from* where the head skeleton point was at time _
3) the average distances moved by the head and tail during the time interval is larger than 2.5% of body length.

Reversal = Percentage of time that a worm performs a reversal (rearward movement)

reversalCount = Number of reversal sessions (periods of rearward movement) that a worm performs during a recording session

reversalDisAve = Mean distance the centroid travels during a reversal session

Body waving

1. Features based on sktp analysis (Part I)

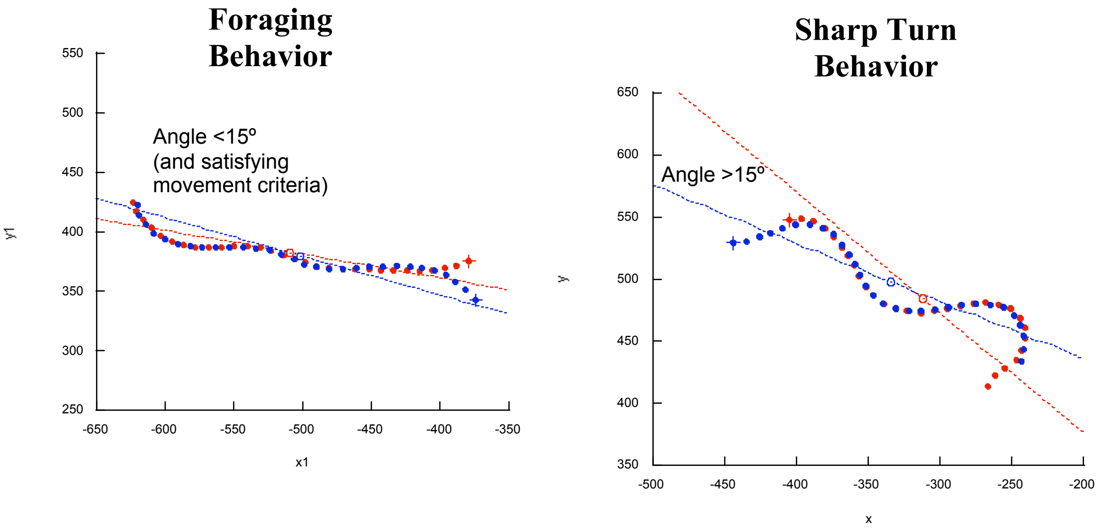

Figure 8 shows the sktp sets, centroids and skewer fits of an N2 animal at two consecutive time points τ (red) and $\tau + 1$ (blue). Left panel shows the animal performing foraging behavior; right panel shows an animal demonstrating sharp turn behavior. When the angle between the skewer fits at times _ and _ + 1 is smaller than 15º, our system identifies foraging behavior provided:

1) the average distance traveled by last 20 skeleton points is less than 2 % of the body length, and
2) the average distance traveled by first 4 skeleton points is larger than 4 times of the average distance traveled by the last 20 skeleton points.

If the angle between the skewer fitting of time _ and that of time _ + 1 is larger than 15º, a sharp turn is counted.

foraging	=	percentage of time that a worm performs foraging behavior (as described above)
turn	=	percentage of time that a worm exhibits sharp turn behavior
foraging angle	=	the angle between skewer fits at all consecutive times _ and _ + 1 when foraging behavior is identified at time _ + 1
foraging distance	=	distance moved by skeleton point P_0 the head skeleton point) between time _ and _ + 1 when a foraging behavior is identified at time _ + 1

2. Features based on sktp analysis (Part II)

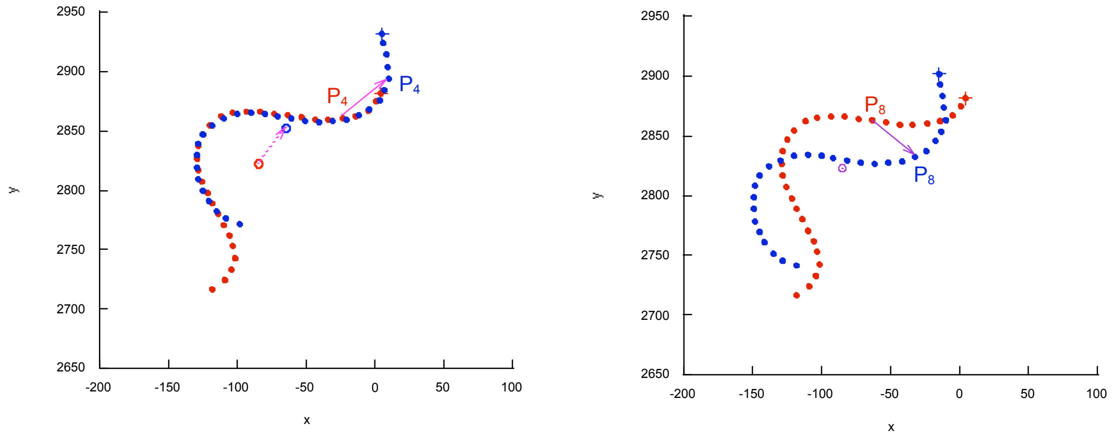

Figure 9 shows the sktp sets and centroids of a forward-moving N2 animal at two consecutive time points τ (red) and $\tau + 1$ (blue). The movement of a specific sktp, P_4, are calculated (left panel). To quantify an aspect of body waving that we call 'pushing' our system compares the patterns of skeleton point sets at consecutive time points by superimposing them on a common centroid (right panel), allowing calculation of individual sktp movement, regardless of whole body movement.

HdTlRatio = Ratio of head movement distance to tail movement distance during a time interval τ to $\tau + 1$

lclSpeed = Distance traveled by the P_4 / Time difference between time τ and $\tau + 1$

Pushing = Ratio of the mean distance all sktp traveled between time τ and $\tau + 1$ (calculated after superimposing sktp set centroids) to the actual distance traveled by the worm's centroid during the same time interval

3. Features based on sktp analysis (Part III)

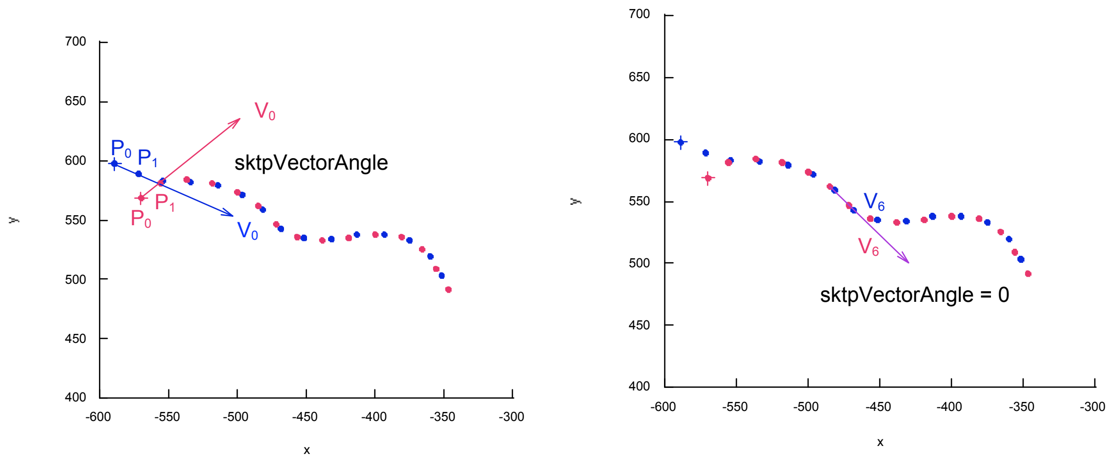

Figure 10 shows two consecutive skeleton points sets for an N2 animal at time τ (in red) and $\tau + 1$ (in blue). Each skeleton point vector, V_i, for example V_0 (shown in left panel), or V_6 (right panel) pivots when the animal navigates.

The angle between adjacent sktp vectors V_i and V_{i+1}, located at skeletonBend[i], is designated as its sktpVectorAngle. Each sktpVectorAngle varies with time as the animal moves. Our system uses the varying sktpVectorAngle to calculate 'flex' and bending frequency ('fre') for each skeletonBend.

We calculate 'flex' as the maximum sktpVectorAngle *difference* (that is, most positive angle minus most negative angle) demonstrated during a time interval, for each skeletonBend.

Likewise, our to extract the bending frequency at each skeletonBend of an animal we apply the *specgram* function provided in The MathWorks' Matlab (R13) Signal Processing toolbox software (http://www.mathworks.com/access/helpdesk/help/toolbox/signal/specgram.html) to compute the discrete-time Fourier transform of the time-varying sktpVectorAngle "signal" with a sliding (32 frame wide) time window. The specgram function yields a representation of the magnitude and phase of the component frequencies of the sktpVectorAngle "signal" for every 32 frame time window. Our system identifies the bending frequency at each skeletonBend (for each time window) as the component frequency with the highest magnitude.

Flex = Maximum sktpVectorAngle difference within each time window of 32 frames. (Calculated for each skeletonBend.)

Fre = The characteristic sktpVectorAngle bending frequency at each skeletonBend from each time window of 32 frames

Additional morphological and behavioral analysis

Our system further evaluates the attributes 'Area' (area of the worm shown in a binary image) and 'GlbSpeed' (centroid speed) by binning the range of values for each attribute, done in the same manner as when creating a histogram. For these evaluations our system uses 12 evenly spaced bins, representing 12 evenly spaced ranges of area or centroid speed.

MostPopularSpeed = Centroid speed range representing the most commonly occurring centroid speeds (GlbSpeed) in a tracking session. (Expressed as the mean of value of the speed range.)

PercentageMPSpeed = Percentage of the time that GlbSpeed values fall into the 'MostPopularSpeed' range.

MostPopularArea = Area value range representing the most commonly occurring worm area in a tracking session. (Expressed as the mean value of the Area value range.)

PercentageMPArea = Percentage of the time that Area values fall into the 'MostPopularArea' range.
