# Supplementary material for: An imaging system for standardized quantitative analysis of C. elegans behavior
Source: BMC Bioinformatics. 2004 Aug 26;5:115. doi: 10.1186/1471-2105-5-115 (PMC517925; doi:10.1186/1471-2105-5-115)
Supplement: Additional File 4 — File list [file 1471-2105-5-115-S4.doc]

The complete file list to compile and run the tracker system.

1. Tracker

closeAVI.h

closeAVI.lib

closeAVI.dll

createAVIWithFilter.h

createAVIWithFilter.lib

createAVIWithFilter.dll

ImageProcessing.c

Imageprocessing.h

wormClassify.c

wormClassify.h

wormClassify.uir

wormDataStruct.c

wormDataStruct.h

wormTracker.prj

WriteFrameAlongDataLib.h

WriteFrameAlongData.lib

WriteFrameAlongDataLib.dll

1. Converter

backBoneAnalysis.c

backboneanalysis.h

backboneDataStruct.h

backboneDataStruct.h

closeAVI.h

closeAVI.lib

closeAVI.dll

ImageProcessing.c

ImageProcessing.h

OpenAVILib.h

OpenAVILib.lib

OpenAVILib.dll

readAVIsFrame.h

readAVIsFrame.lib

readAVIsFrame.dll

wormClassify.c

wormClassify.h

wormClassify.uir

wormConverter.prj

1. Lineup

backBoneAnalysis.c

backBoneAnalysis.h

backBoneData.c

backBoneData.h

backbonelineup.c

backboneLineup.h

wormClassify.c

wormClassify.h

wormClassify.uir

1. miner

backboneDataStruct.c

backboneDataStruct.h

contraction4lib.h

contraction4lib.lib

contraction4lib.dll

miner.c

miner.h

talkWithDatabase.c

talkWithDatabase.h

trackslib.h

trackslib.lib

trackslib.lib

WormDataManipulation.c

WormDataManipulation.h

wormMiner.c

wormMiner.h

wormMiner.uir

1. the database file

wormBehavior.mdb

2. Software require to compile and run the tracker system

National Instrument Developer Suite (Professional) (NI product number 77902-03)

NI Vision Development Module for Measurement Studio (NI product number 77860-03)

3. License need to run the tracker system only

NI IMAQ Vision Deployment License (NI catalog number 778044-00)
